# Ameliorative effects of *Achillea millefolium* inflorescences alcoholic extract on nicotine-induced reproductive toxicity in male rat: Apoptotic and biochemical evidences 

**Published:** 2017-06-15

**Authors:** Ameneh Hasanzadeh Khosh, Shapour Hasanzadeh, Ali Shalizar Jalali

**Affiliations:** *Department of Basic Sciences, Faculty of Veterinary Medicine, Urmia University, Urmia, Iran.*

**Keywords:** *Achillea millefolium*, Nicotine, Oxidative Stress, Rat, Sperm

## Abstract

Nicotine (NIC) adversely influences male reproductive system*. Achillea millefolium *(Achm) as a medicinal plant is highly regarded for its antioxidant and anti-inflammatory properties. The present study was conducted to assess whether Achm inflorescences alcoholic extract could serve as a protective agent against reproductive toxicity in NIC-exposed male rats. Adult male rats were randomly divided into six groups. Two groups received NIC at doses of 0.20 and 0.40 mg kg^-1 ^per day in 0.50 mL sterile distilled water for 48 days intraperitoneally, respectively. The further two groups received NIC at doses of 0.20 and 0.40 mg kg^-1^ per day in 0.50 mL sterile distilled water for intraperitoneally along with Achm extract at a dose of 1.20 g kg^-1 ^per day in 1 mL sterile distilled water orally for 48 days, respectively. A vehicle treated control group and an Achm-only treated group were also included. The NIC-exposed groups showed significant reductions in epididymal sperm count, motility, viability and serum levels of FSH, LH and testosterone as well as testicular antioxidant capacity. Moreover, the incidence of apoptosis and abnormality in spermatozoa along with testicular malondialdehyde and total nitrite levels were significantly higher in NIC-treated rats. The above-mentioned parameters were restored to near normal levels by Achm co-administration. These findings indicated that Achm may partially be protective against NIC-induced testicular toxicity.

## Introduction

The adverse effects of nicotine (NIC) on the male reproductive system in the different species of mammals have been recognized. Cigarette smoking has been found to be a significant risk factor for decreased semen quality in adult men.^1^ It has been shown that laboratory animals exposures to NIC lead to testicular weight reduction, elevation of sperm abnormalities and atrophy of accessory glands, epididymis and vas deferens.^2^

It is well documented that dose and timing of NIC exposures in rats correlate with destructive changes in testicular tissue.^3^ Moreover; *in vivo* studies have revealed that NIC is capable of inducing apoptosis in Leydig cells.^4^

Despite fact that the mechanisms of NIC-induced side effects are unclear, but more likely it causes acetylcholine and some other neurotransmitters release and induces disturbances in the structural and functional aspects of metabolic systems in body.^5^

Growing evidence indicates that antioxidants can prevent not only the decrease in sperm quality, but also enhance its functionality.^6^ Reportedly, the antioxidants can increase pregnancy rates through reduction in sperms DNA damages and apoptosis.^7^

*Achillea millefolium *(Achm), a medicinal plant used by many cultures for over 3000 years, have been shown to have anti-inflammatory, antitumor, antimicrobial, liver protective and antioxidant properties.^8^ The main compounds found in this plant include volatile oils, poly-phenol, flavonoids, sesquiterpene lactones, betaine, polyacetylenes, resins and tannins. ^9^

It has also been reported that Achm extract, as a potent repro-protective compound, can help to prevent oxidative stress-evoked testicular toxicities in animal models. ^8^^, ^^10^

In the line with that, the present study was carried out in order to uncover the new aspects from the possible mechanism(s), by which the NIC is able to adversely impact on sperms and the ameliorative effects of Achm for these noxious effects. For this purpose, the quantitative as well as qualitative and apoptotic changes of rat epididymal sperms and protective potentials of Achm alcoholic extract against these changes were aimed to investigate.

## Materials and Methods

**Plant material. **The Achm was harvested from its natural habitat around the city of Urmia in West Azerbaijan province, northwest Iran during the flowering season (between May and July). The identification of collected plants was confirmed scientifically at the research laboratories of the Department of Agriculture of West Azerbaijan province.

**Preparation of the alcoholic extract. **The alcoholic extract of dried inflorescences of the plant was prepared by infusion of the finely dried material in methanol 70%, at 20 ˚C (1:10, w/v) for 36 hr.^11^ The infusion was filtered and concentrated with rotary machine. The concentrated and completely dried extract was diluted in distilled water immediately before use. 

**Nicotine. **Liquid NIC (1-methyl-2-(3-pyridyl) pyrolidine > 99% (GS) was procured from Sigma-Aldrich (St. Louis, USA) and diluted in sterile distilled water immediately before use.

**Animal model.** In this study, thirty-six adult sexually mature male (10 weeks of age weighing 225.32 ± 4.48 g) albino rats of Wistar strain were obtained from authorized laboratory animal breeding center (The Laboratory Animal House, Urmia University, Urmia, Iran). They were housed in a specific pathogen-free environment under standard conditions of temperature (25.00 ± 2.00 ˚C), relative humidity (50.00 ± 10.00%) and light (12 hr light/12 hr dark), fed with a standard pellet diet and had free access to water. Body weights were recorded weekly during the experiment. Clinical and behavioral observations were also recorded throughout the study. Animal work was conducted in compliance with guidelines for the humane care and use of laboratory animals using protocols approved by the Urmia University.

**Experimental protocol. **Following 10 days of acclimatization to the new environment, the rats were randomly divided into six groups, each comprises of six animals. Group 1 was provided as control receiving 1 and 0.50 mL sterile distilled water orally and intraperitoneally respectively throughout the experiment. Group 2 was provided as Achm alcoholic extract control receiving the extract (1.20 g kg^-1^ per day) dissolved in 1 mL sterile distilled water orally. Group 3 and 4 were received NIC dissolved in 0.50 mL sterile distilled water at doses of 0.20 and 0.40 mg kg^-1^ per day; IP, respectively and groups 5 and 6 were received NIC at doses of 0.20 and 0.40 mg kg^-1^ per day dissolved in 0.50 mL sterile distilled water respectively through IP route along with Achm alcoholic extract (1.20 mg kg^-1^ per day) dissolved in 1 mL sterile distilled water orally for a period of 48 days. The etiquettes for this study including doses and duration of NIC and Achm treatments were all designed according to previous studies.^12^

**Sampling. **The animals were euthanized by CO_2_ exposure following anesthesia with ketamine (75 mg kg^-1^; IP) 24 hr after the last treatment. Testes and epididymides were rapidly dissected out, cleared of adhering connective tissue and weighed on a Mettler Basbal scale (Delta Range, Tokyo, Japan). The testes were preserved in liquid nitrogen for biochemical evaluations. 

**Sperm motility. **In order to assess the sperm motility, one caudal epididymis was placed in 1 mL of rat 1-cell embryo culture medium (mR1ECM). Cauda epidydimis was cut into two to three pieces and incubated at 37 ˚C for 10 min with 5% CO_2_ incubator to allow sperms to swim out of the epididymal tubules. One drop of sperm suspension was placed on a microscope slide and a cover slip was placed over the droplet. At least 10 microscopic fields were observed at 400× magnification using a phase contrast microscope and the percentage of motile sperms was evaluated microscopically within 2 to 4 min of their isolation from the epididymides and expressed as a percentage of motile sperm of the total sperm counted.^13^

**Epidydimal sperm count. **Sperm concentration was determined by hemocytometer (HBG, Germany). After dilution of epididymal sperm to 1:20 in mR1ECM medium, approximately 10 μL of this diluted specimen were transferred to each of the counting chambers of the hemocytometer, allowed to stand for 5 min in a humid chamber to prevent drying. The cells in the sediment during this time were counted up with a light microscope at 400×. The sperm count was expressed as the number of sperm per mL (n × 50000 × d), where, n is counted sperms and d is reverse of dilution solution.^14^

**Sperm viability. **20 μL of sperm suspension were mixed with equal volumes of 0.05% Eosin-Y. After 2 min incubation at room temperature, slides were viewed by bright-field microscope with 400× magnification. Dead sperms appeared pink and live sperms were not stained. Two hundred sperms were counted for each sample and viability percentages were calculated.^13^

**Fig. 1:**
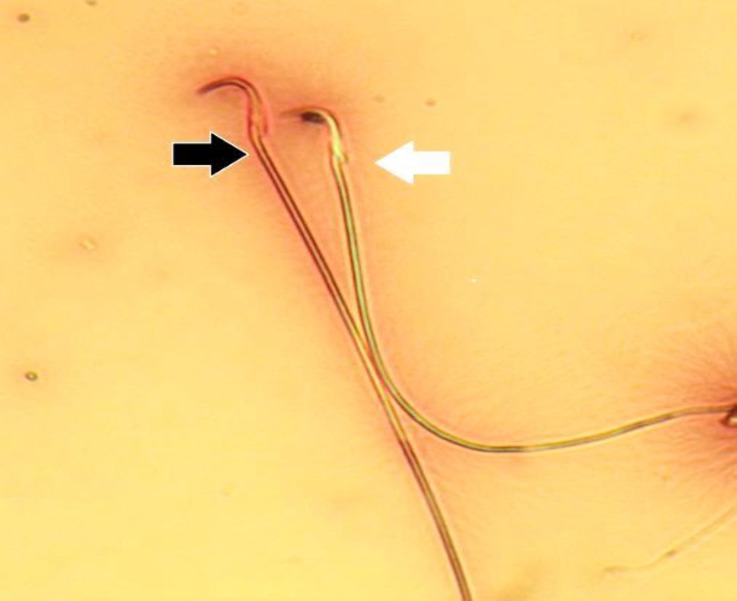
Photomicrograph of epididymal sperms. Dead sperm (black arrow) appears pink and live sperm (white arrow) is not stained (Eosin-nigrosin, 1000×).

**Teratozoospermia Index (TZI). **Sperm smears were prepared on clean and grease free slides, allowed to air-dry overnight, stained with 1% eosin-Y/5% nigrosin and examined at 400× magnification for morphological abnormalities (Fig. 1). The TZI is defined as the number of abnormalities present per abnormal spermatozoon. Each abnormal spermatozoon can have one to four ab-normalities including head, neck/mid piece and tail defects or presence of cytoplasmic residues. The spermatozoa are recorded as normal or abnormal and distributed into specific groups (head, neck/mid piece and tail defects or cytoplasmic residues groups). The total number of abnormalities is then added together and divided by the number of abnormal spermatozoa.^15^

**Fig. 2:**
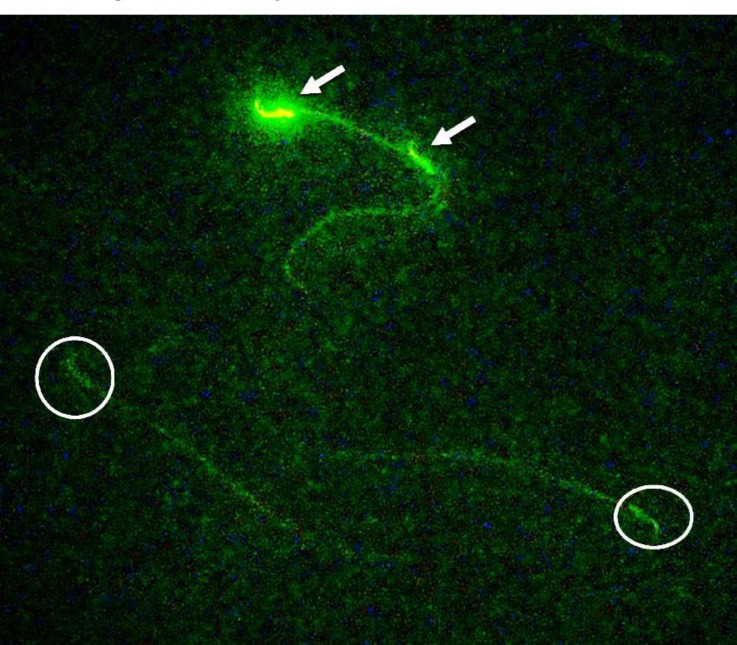
Photomicrograph of epididymal sperms. Apoptotic sperms (white arrows) exhibit green fluorescence, whilst non-apoptotic sperms (circles) are unstained (Annexin-V, 800

**Sperm apoptosis. **To detect phosphatidylserine translocation, the Annexin-V FITC apoptosis detection kit was used (BD Biosciences Pharmingen, San Diego, USA). To perform this analysis, semen samples containing 1 × 10^6^ spermatozoa were centrifuged at 5000 rpm for 6 min and re-suspended in equal volume of HEPES-buffered saline. Semen suspension was mixed with 100 μL annexin-V/fluorescein isothiocyanate solution and incubated for 15 min at room temperature. Staining with annexin V was checked under fluorescent microscope using 488 nm wave-length filters. Sperm with disordered membrane exhibited green fluorescence, whilst live sperm was unstained (Fig. 2). Apoptotic index was defined as the number of apoptotic annexin V-positive sperm cells per 100 spermatozoa.^16^

**Assessment of testicular total antioxidant capacity (TAC). **To assess beneficial effects of Achm alcoholic extract, testicular tissue TAC was measured. The assay is based on the assessment of ferric reduction antioxidant power (FRAP).^17^ In this method, at low pH which was achieved by adding of acetate buffer (300 mMol L^-1^, pH 3.60), reduction of Fe^III^-TPTZ complex to the ferrous form produces an intensive blue color that could be measured at 593 nm. Aqueous solution of Fe^II^ and appropriate concentrations of freshly prepared ascorbic acid were used as blank and standard solutions, respectively. The TAC was expressed as nMol mg^-1^ protein of the samples. The protein content of the samples was measured according to the Lowry method.^18^

**Measurement of testicular total thiol molecules (TTM). **In order to evaluate the total sulfhydryl levels in the testes, 3.00 to 4.00 g of testicular tissue samples were mixed in cold potassium chloride. The KCL 1% homo-genized mixture was centrifuged for 10 min at 3000 rpm. 2.00 mL of the supernatant of homogenized tissue were added to 6.00 mL of Tris-EDTA buffer and then 40 mL DTNB reagent was added. Finally, with methanol solution it was brought to 4 mL. Following 15 min incubation at room temperature, samples were centrifuged again for 10 min in 3000 rpm. Absorption at 412 nm was evaluated by a spectrophotometer. The total amounts of thiol molecules were expressed as nMol mg^-1^ protein.^19^

**Measurement of testicular malondialdehyde (MDA). **The MDA was measured with thiobarbituric acid at 532 nm in a spectrophotometer (Biochrom, Cambridge, UK) as described previously. ^20^ Results were expressed as nMol per mg protein.

**Determination of total nitrite content (TNC) in testicular tissue. **The total oxidation products of nitric oxide (NO) metabolism (NOˉ_2_/NOˉ_3_) of testicular tissue were assessed using a Griess reagent. The Griess reagent consists of sulfanilamide (SULF) and N-(1-Naphthyl) ethylenediamine dihydrochloride (NEDD). The testicular tissue (0.20 to 0.30 g) was mixed with zinc sulfate solution (Sigma, USA), homogenized and then, centrifuged at 12,000 *g* for 10 min. Aliquots (300 μL) of the clear supernatant were mixed with Griess reagents including 300 μL SULF (2% w/v, Sigma, USA) in 5% HCl and 300 μL NEDD (0.10% w/v, Sigma, USA) in H_2_O in a test tube, while for reduction of nitrate to nitrite, 300 μL saturated solutions of vanadium (III) chloride (VCl3; Sigma, USA) in 1 M HCl were added and incubated for 2 hr at 30 ˚C in the dark. Due to this reaction, NO is rapidly converted to more sustainable nitrite which in acidic pH is converted to HNO_2_. The reaction of HNO_2_ with SULF causes releasing of diazunium salt. This salt following reaction with the reagent provides Azo color that can be evaluated by a spectrophotometer at 540 nm. Results were expressed as nMol per mg of protein.^21^

**Hormonal assays.** Serum concentrations of FSH and LH were assayed by electrochemiluminescence immuno-assay (Monobind Inc., Lake Forest, USA) as well as testosterone level (Roche Diagnostics GmbH, Kiel, Germany). 

**Statistical analysis. **Results are expressed as mean ± SE. Differences between groups were assessed by one way analysis of variance using the SPSS software package for Windows (version 18; SPSS Inc., Chicago, USA). Statistical significance between groups was determined by Tukey multiple comparison post hoc test and the *P*-values less than 0.05 were considered to be statistically significant.

## Results

**Body and testicular weights. **The body weight decreased by both NIC doses; Achm restored this parameter towards controls. The NIC exposures were significantly decreased body weight, especially in higher dose NIC group, while Achm co-administration significantly improved body weights in NIC-Treated animals towards controls (Table 1).

**Epididymal sperm characteristics. **In the groups which received low and high doses of the NIC compared to the controls, significant reductions (*p* < 0.05) in sperm content, motility and viability were present, meanwhile significant increases (*p* < 0.05) were observed in abnormal sperms percentages. Administration of alcoholic extract of Achm led to increase in sperm concentration, motility and viability along with sperm abnormality reduction (Table 2). 

Administrations of low and high doses of NIC significantly (*p* < 0.05) increased teratozoospermia, meanwhile administration of alcoholic extract of Achm led to significant (*p* < 0.05) decreases in TZI (Table 2).

**Fig. 3:**
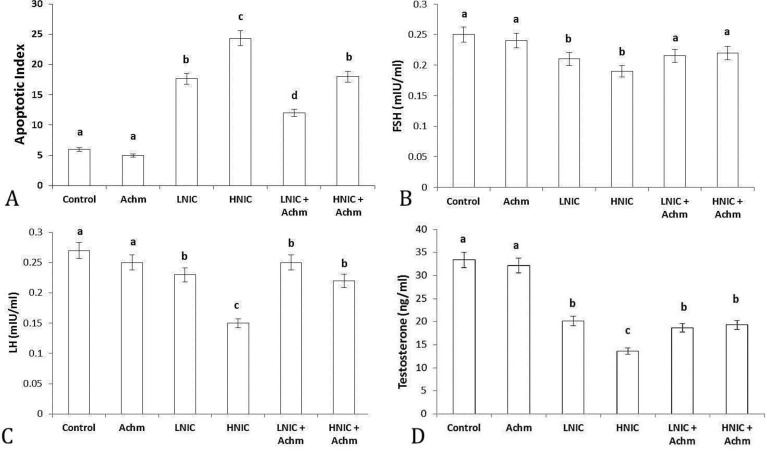
Effect of nicotine and *Achillea millefolium* alcoholic extract on epididymal sperm apoptosis (A) and serum concentrations of FSH (B), LH (C) and testosterone (D) in all experimental groups. Achm; *Achillea millefolium*, LNIC; Low dose nicotine, HNIC; High dose nicotine.

^abcd^ Different superscripts denote statistical significance at *p* < 0.05.

**Epididymal sperm apoptosis. **Administration of low and high doses of the NIC significantly (*p* < 0.05) increased the incidence of sperm apoptosis, whereas administration of alcoholic extract of Achm led to significant (*p *< 0.05) decreases in this event (Fig. 3A).

**Hormonal analyses. **The serum levels of FSH (Fig. 3B), LH (Fig. 3C) and testosterone (Fig. 3D) were decreased in NIC-treated groups. Simultaneous administration of Achm along with NIC restored the aforementioned parameters towards control values**.**

**Testicular TTM. **The TTM analyses in the testicular tissues of different groups compared with the controls have shown that this parameter in low dose NIC receiving group does not have significant difference, but in high dose NIC-treated group TTM levels were decreased in comparison with control group. Administration of alcoholic extract of Achm along with NIC caused no significant changes in this parameter as compared to NIC groups (Table 3).

**Testicular TAC. **The levels of TAC in testes were decreased in NIC receiving groups in comparison to controls, but restored in groups that received low and high doses of NIC along with Achm alcoholic extract (Table 3). 

**Table 1:** Effect of nicotine and *Achillea millefolium *alcoholic extract on body and organ weight (n = 6).

**Variables**	**Control **	**Achm **	**LNIC **	** HNIC **	**LNIC +** ** Achm**	**HNIC +** ** Achm **
**Body weight (g) **	240.00 ± 12.20	235.00 ± 14.60^a^	219.66 ± 8.60^b^	200.00 ± 22.20^b^	226.66 ± 11.3^a^	218.00±7.90^a^
**Absolute testes + epidymides weight (g)**	2.21± 0.14	2.11± 0.21^a^	2.32± 0.02^a^	2.29± 0.12^a^	1.89± 0.03^b^	2.45± 0.20^a^
**Relative testes + epidymides weight (%)**	0.98 ± 0.52	0.97 ± 0.16^a^	1.01 ± 0.01^a^	0.79± 0.03^a^	0.72± 0.3^a^	0.75± 0.01^a^

Achm: *Achille amillefolium*; LNIC: Low dose nicotine; HNIC: High dose nicotine.

ab Different superscripts denote statistical significance at *p* < 0.05.

**Table 2:** Effect of nicotine and *Achillea millefolium *alcoholic extract on epididymal sperm characteristics (n = 6).

**Variables**	**Control **	**Achm **	**LNIC **	** HNIC **	**LNIC +** ** Achm**	**HNIC +** ** Achm **
**Sperm count (10** ^6^ ** mL** ^-1^ **)**	48.00 ± 1.15	51.00 ± 0.57^a^	35.66 ± 0.88^b^	25.66 ± 0.88^c^	40.66 ± 0.88^d^	34.33 ± 1.45^b^
**Sperm viability (%)**	90.16 ± 0.22	91.66 ± 0.30^a^	67.58 ± 0.44^b^	49.50 ± 0.43^c^	73.75 ± 0.28^d^	61.50 ± 0.66^e^
**Sperm motility (%)**	80.97 ± 0.61	84.60 ± 0.81^a^	63.60 ± 1.03^b^	41.12 ± 0.93^c^	71.60 ± 0.92^d^	53.62 ± 0.56^e^
**Abnormal sperms (%)**	8.25 ± 0.14	7.00 ± 0.14^a^	18.66 ± 0.30^b^	27.58 ± 0.44^c^	13.00 ± 0.14^d^	19.83 ± 0.36^b^
**Teratozoospermia Index**	1.08 ± 0.04	1.04 ± 0.04^a^	1.41 ± 0.00^b^	1.77 ± 0.03^c^	1.27 ± 0.02^d^	1.43 ± 0.01^b^

Achm: *Achille amillefolium*; LNIC: Low dose nicotine; HNIC: High dose nicotine.

**abcde:** Different superscripts denote statistical significance at *p* < 0.05.

**Table 3:** Effect of nicotine and *Achillea millefolium *alcoholic extract on testicular tissue biochemical parameters (n = 6).

**Variables**	**Control**	**Achm **	**LNIC **	** HNIC **	**LNIC +** ** Achm **	**HNIC +** ** Achm **
**MDA (nmol mg** ^-1^ **)**	2.40 ± 0.50	2.08 ± 0.30^a^	7.20 ± 0.20^b^	11.20 ± 0.60^b^	2.20 ± 0.20^c^	5.80 ± 0.60^c^
**TNC (nmol mg** ^-1^ ** )**	6.74 ± 1.20	5.90 ± 0.20^a^	105.80 ± 2.30^b^	90.70 ± 2.30^b^	11.20 ± 4.01^c^	11.20 ± 4.03^c^
**TAC (nmol mg** ^-1^ ** )**	0.13 ± 0.00	0.13 ± 0.00	0.10 ± 0.00^b^	0.10 ± 0.00^b^	0.12 ± 0.0^c^	0.12 ± 0.00^c^
**TTM (nmol mg** ^-1^ ** )**	1.70 ± 0.51	1.80 ± 0.50	1.40 ± 0.30^a^	1.01 ± 0.02^a^	1.30 ± 0.50^a^	1.10 ± 0.40 a

Achm: *Achille amillefolium*; LNIC: Low dose nicotine; HNIC: High dose nicotine, MDA; Malondialdehyde, TNC; Total nitrite content, TAC; Total antioxidant capacity, TTM; Total thiol molecules.

abc Different superscripts denote statistical significance at *p* < 0.05.

**Testicular MDA. **In rats received low and high doses of NIC, the amounts of MDA production were significantly (*p *< 0.05) higher than control group, but in groups receiving low and high doses of NIC along with Achm, MDA level was significantly (*p* < 0.05) decreased in comparison to the NIC-only receiving groups (Table 3).

**Testicular TNC. **The NIC in low and high doses caused significant increases (*p *< 0.05) in TNC compared to control group, while Achm co-administration improved this parameter compared to the groups receiving low and high doses of NIC.

## Discussion

 The NIC is an active alkaloid and addictive material and its effects are extensive on male reproductive system and fertility.^22^^,^^23^ According to the results of this study, NIC at both doses of 0.20 and 0.40 mg per kg per day caused reductions in epididymal sperm quality and quantity and increased apoptotic changes in epididymal sperms in rats.

 One of the reasons for initiation of sperm apoptosis is deficit of energy supply. The NIC acts on spermatocytes, spermatids and spermatozoa through effects on the acetylcholine receptors of these cells.^24^ On the cell membranes, NIC induces lipid peroxidation of the unsaturated fatty acids and produces reactive oxygen species (ROS). The ROS inhibit vital enzymes such as glucose-6-phospahte dehydrogenase which plays crucial role in regulation of glucose level in the cell.^25^ Based on this concept; the sperms come across with energy deficit.

According to previous reports, permeability of blood vessels increases following increase in NO levels which occurs following NIC use, bringing about hyperemia and edema in the testicular tissue.^26^ This condition can lead to disturbances in nourishment of the germinal epithelium by Sertoli cells. It has been reported that NIC causes disturbances in function of Leydig cells and brings about decreases in the level of testosterone.^4^ The findings of present study are in accordance to the results of previous report in which the NIC was administered at doses of 0.10, 0.20 and 0.40 mg kg^-1^ per day.^12^ Due to the importance of Leydig cells in secretion of testosterone which plays key role in spermatogenesis, destruction of these cells can be a major cause of sperm apoptosis and death elevation.

The NIC easily passes through cell membrane and combines with cytoplasmic constituents and causes disturbances in the process of cells division. The acetylcholine and NIC receptors not only are present in nerve and muscular cells, but also in most of non-nervous cells such as cells in reproductive system.^27^ Therefore, NIC by exerting effects on acetylcholine receptors causes disturbances in the functions of cells and induces sperm apoptosis and abnormality. In this study, the Annexin-V staining technique revealed sperm apoptosis elevation following NIC administration and restoration of this process by Achm alcoholic extract co-administration. 

Further, NIC causes insulin resistance and leads to increase in its secretion resulting in disturbances in glucose transport into cells in a dose dependent manner, causing instability in sperm energy cycle and finally apoptosis.^28^

**Fig. 4.**
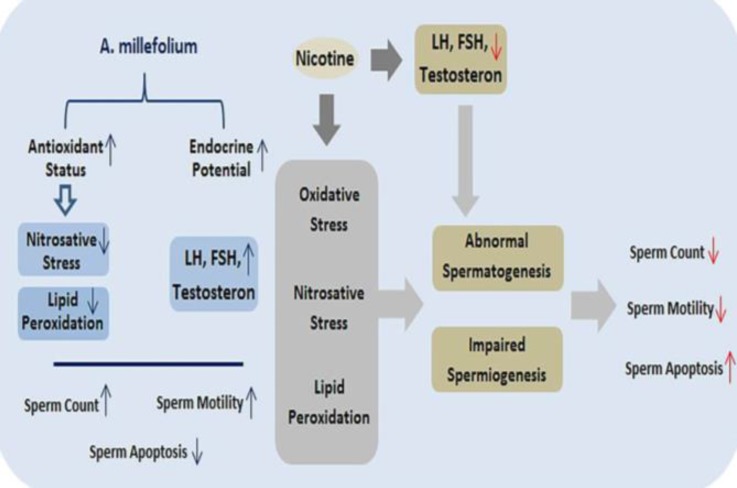
Nicotine-induced reproductive toxicities and ameliorative effect of* Achillea millefolium *alcoholic extract; Nicotine reduces gonadotropins secretions and induces oxidative and nitrosative stresses which are negatively affect spermatogenesis mainly via provoking lipid peroxidation. Impaired spermatogenesis in turn results in diminished sperm quantity and quality. However, *Achillea millefolium *alcoholic extract ameliorates nicotine-induced reprotoxicities through testicular antioxidant status improvement and biochemical stresses inhibition

It has been shown that NIC causes increase in the levels of the ROS and MDA in the testicular tissue.^29^Confirming previous findings, this study also demonstrated that NIC causes oxidative stress in rat testicular tissue. One can conclude that sperm concentration reduction in rats received low and high doses of NIC is associated with oxidative stress evoked germinal cells destruction, apoptosis induction and sperm damage (Fig. 4).

In this study, the reduction in sperm motility was seen in a dose dependent manner which is in conformity with previous reports.^30^^,^^31^

It was found that NIC can cause DNA damages in testicular tissue^2^. Further, reduced testosterone level and DNA damage could be causative factors for sperm abnormality and apoptosis.^16^ In line with that, it seems that observed NIC-related reprotoxicity in this study can be associated with reproductive tissue disorganization,^32^ DNA damages as well as androgenesis suppression. 

Beneficial roles of various antioxidants against destructive effects of NIC in male reproductive system have been shown previously.^28^^,^^33^^,^^34^ In this study, alcoholic extract of Achm also showed remarkable protection against deleterious effects of NIC in reproductive system probably through oxidative stress inhibition.^8^ It is well known that significant antioxidant activities of Achm is due to the presence of phenolic and flavonoid contents.^35^ Accordingly, Achm protective effects against cyclophosphamide-induced testicular toxicities as well as spermatotoxicities of doxorubicin have been revealed previously. ^8^^,^^10^^,^^36^

Taken together, it can be concluded that NIC induces sperm impairment in a dose dependent manner possibly via oxidative stress induction, testicular NO level elevation and testicular TAC level reduction. Notably, concurrent administration of Achm alcoholic extract attenuated NIC-induced reproductive toxicities probably through scavenging ROS and strengthening antioxidant defense system in reproductive tissues (Fig. 4).
